# How did outdoor biking and walking change during COVID-19?: A case study of three U.S. cities

**DOI:** 10.1371/journal.pone.0245514

**Published:** 2021-01-20

**Authors:** Annie Doubleday, Youngjun Choe, Tania Busch Isaksen, Scott Miles, Nicole A. Errett

**Affiliations:** 1 Department of Environmental and Occupational Health Sciences, School of Public Health, University of Washington, Seattle, WA, United States of America; 2 Department of Industrial and Systems Engineering, University of Washington, Seattle, WA, United States of America; 3 Department of Health Services, School of Public Health, University of Washington, Seattle, WA, United States of America; 4 Department of Human Centered Design and Engineering, University of Washington, Seattle, WA, United States of America; Drexel University School of Public Health, UNITED STATES

## Abstract

A growing body of literature suggests that restrictive public health measures implemented to control COVID-19 have had negative impacts on physical activity. We examined how Stay Home orders in Houston, New York City, and Seattle impacted outdoor physical activity patterns, measured by daily bicycle and pedestrian count data. We assessed changes in activity levels between the period before and during Stay Home orders. Across all three cities, we found significant changes in bicycle and pedestrian counts from the period before to the period during Stay Home orders. The direction of change varied by location, likely due to differing local contexts and outbreak progression. These results can inform policy around the use of outdoor public infrastructure as the COVID-19 pandemic continues.

## Introduction

During Stay Home orders over the course of the COVID-19 pandemic, outdoor public infrastructure, including parks and walking and biking trails, are some of the few low-risk spaces individuals can easily access outside their homes. However, outdoor public infrastructure use at the community level during the pandemic is not well-understood beyond limited self-reported data. A better understanding of these patterns could serve as an indicator of how communities are adapting throughout the COVID-19 pandemic, and in future public health disasters.

Prior research has suggested that physical activity [1] and time spent outside [2] are both associated with increased wellbeing. COVID-19-specific studies suggest that a decrease in physical activity may increase the risk of cardiovascular disease [3] and metabolic disorders [4], among other health impacts. A recent study in China utilizing survey data also found that physical activity during the pandemic mitigated negative emotions in university students [5], suggesting impacts beyond physical health.

A small but growing body of literature on behavior change during the COVID-19 pandemic provides evidence of population-level physical activity changes. One recent descriptive study found an overall decrease in step counts following Stay Home orders, although the authors report variability within and between countries [6]. A study of 1,000 Canadian adults found pandemic-related changes in activity levels related to pre-pandemic physical activity levels. Specifically, they found participants who were inactive before the pandemic and engaged in more outdoor physical activity during the pandemic had lower anxiety levels than those who engaged in less outdoor physical activity [7]. Another study in Canada of 1,500 children and youth found that the percent of children and youth meeting national activity guidelines was much lower during the pandemic period than in previous surveys [8]. Additionally, a study reporting results of the first 1,000 participants in the "Effects of Home Confinement on Multiple Lifestyle Behaviours during the COVID-19 Outbreak" (ECLB-COVID19) international survey found that policies and orders keeping individuals at home reduced physical activity levels [9].

These studies provide insights on physical activity changes during the COVID-19 pandemic and their impacts on wellbeing. However, they rely on self-reported data, highlighting the need for research utilizing objective, real-time measures of physical activity to better understand the impacts of the Stay Home orders on physical activity and wellbeing. Since March 2020, jurisdictions across the U.S. have implemented restrictive public health measures to help curb the spread of COVID-19 [10]. These measures include banning mass gatherings, school closures, Stay Home orders, and state and local park closures. Restrictions in Houston, New York City, and Seattle, the focus of this analysis, differed and included Stay Home orders, park closures, closures of parking lots at major city parks, and opening of several miles of streets to pedestrians and bicycles to encourage safe outdoor physical activity (Table 1). However, it is not clear how community members utilize these spaces and how these restrictions have impacted outdoor physical activity patterns.

**Table 1:** List of restrictive public health measures by location.

Measure	Houston	New York City	Seattle
Ban on mass gatherings	3/13/20	3/12/20	3/11/20
School closures	3/13/20	3/16/20	3/13/20
Bars and restaurants closed	3/16/20	3/16/20	3/16/20
Closure of non-essential businesses	3/24/20	3/22/20	3/23/20
Stay Home order in effect	3/24/20	3/22/20	3/23/20
First state or local park closures/restrictions	3/25/20	3/24/20	3/25/20
Limited business openings	5/1/20	5/15/20	5/5/20
Stay Home order lifted	6/10/20	6/13/20	6/1/20

Source: Raifman J, Nocka K, Jones D, Bor J, Lipson S, Jay J, and Chan P. (2020). "COVID-19 US state policy database." Available at: www.tinyurl.com/statepolicies.

This study utilizes daily bicycle and pedestrian counts from Houston, New York City, and Seattle as a proxy for outdoor physical activity. Bicycle and pedestrian counts do not rely on self-reported information and have been collected continuously in Seattle since 2014 [11], in New York City since 2016 [12], and in Houston since 2014 [13]. These counts continue to be collected daily during the pandemic, serving as a reliable metric to assess changes in behavior and outdoor activity patterns across the pre-, during, and post-Stay Home order periods. Additionally, much of the data is provided in near-real-time or reported monthly, aiding in rapid assessment of activity change to inform decision-making around policies impacting outdoor public infrastructure use. We chose these three cities for our analysis because they represent three distinct regions of the U.S., each city had at least one major COVID outbreak during the study period (March—July), and each city had different responses to the pandemic.

This exploratory study aims to examine how the Stay Home orders in Houston, New York City, and Seattle impacted outdoor physical activity patterns, as measured by daily bicycle and pedestrian count data.

## Methods

**Fig 1:**
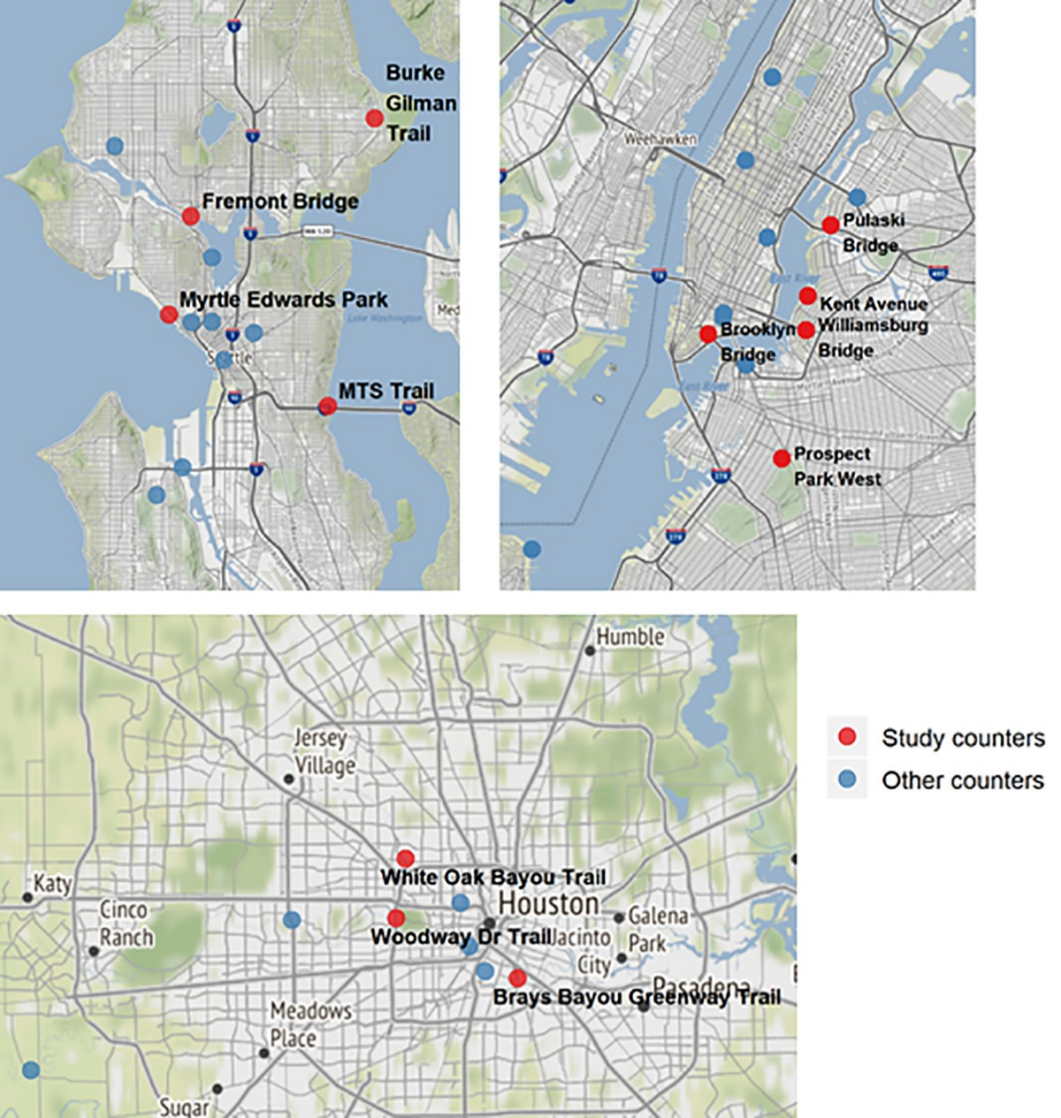
**Location of bicycle counters in Seattle (top left) [11], New York City (top right) [12], and Houston (bottom) [13].** Study counters (in red) are the counters included in the analysis, and other counters (in blue) are all other bicycle/pedestrian counters in each city not included in the analysis due to incomplete data. Base maps and data from © OpenStreetMap contributors and OpenStreetMap Foundation. Contains information from OpenStreetMap and OpenStreetMap Foundation, which is made available under the Open Database License.

We downloaded the publicly available bicycle and pedestrian count data collected by the Seattle Department of Transportation, the New York City Department of Transportation, and the 15-minute level count data from the Texas Department of Transportation for the Houston area [11–13]. In all three jurisdictions, count data are collected by counters placed near bike lanes and bike greenways. According to the publicly available information, the pandemic has not disrupted their respective count programs [11–13]. In Houston, data were only available through May 2020, in New York City, data were available through June 2020, and in Seattle, data were available through August 2020. In this analysis, we focused on a handful of counters in each city that were relatively well distributed across the geographic region, and that had complete data for the entire period including four counters in Seattle, five counters in New York City, and three counters in Houston (Fig 1). Counters with disproportionately missing data for the period were excluded, resulting in incomplete geographic coverage of each region.

We aggregated the count data by day and counter location to yield daily bicycle and pedestrian counts at each counter. All three counters in Houston, none in New York City, and two counters in Seattle collected pedestrian counts over the same period. We focused our analysis on the impact of each area's Stay Home order, which was declared on March 23, 2020 in Washington, March 22, 2020 in New York, and March 24, 2020 in Harris County, Texas [10].

To control for extraneous variables and more accurately assess the impact of the Stay Home orders on physical activity in Houston, New York City, and Seattle, we created a series of linear regression models with bicycle count data and separately with pedestrian count data from January to May, June, or August 2019, depending on data availability in 2020 by city, modeling count data over time. Our choice of linear regression models (as opposed to generalized linear models, for example, with the negative-binomial distribution) was because 1) relatively large daily counts made the use of a discrete distribution optional and 2) residuals generally satisfied the normality assumption according to Q-Q plots. For each counter, we regressed daily counts on various extraneous variables that may influence the counts, such as maximum daily temperature, daily precipitation, day of the week, and week of the year. Each regression model was fitted to the 2019 data to predict daily counts in 2020 using the observed predictor values in 2020. In New York City, we decided that fitting a model to the 2019 data to predict the 2020 pre-Stay Home order data would not give us an accurate measure of the model's predictive power. This decision is based on observed increasing bicycle activity in the month before the Stay Home order (S1 Fig) [14]. Media reports speculated that this may be the result of more New Yorkers commuting by bike instead of the crowded subway due to COVID-19 concerns [14]. Instead, we created regression models with 2018 data, then measured its predictive power on the 2019 data before selecting the best model to predict on the 2020 data.

We chose the prediction model with the lowest root-mean-squared error (RMSE) across the majority of counters for the pre-Stay Home order 2020 data for the final analyses. For consistency across locations, we included the following variables in the final prediction model for all counters, due to the relatively low RMSE across most counters: maximum daily temperature, daily precipitation, and day of the week. Maximum daily temperature and total daily precipitation were drawn from Weather Underground's historical data from a major airport in the region, and were coded as continuous variables [15]. Day of the week was coded as a factor variable in the models. Note that we chose the linear regression model for its good interpretability and predictive performance that allows controlling for the extraneous variables reasonably well. More complex predictive models may yield better RMSEs, but building such models is out of the scope of this paper because the current models already predict pre-Stay Home order trends with enough accuracy (*R*^2^ > 50% generally across all counter locations) to quantify how observed post-Stay Home order trends deviate from the trends predicted if there were not a Stay Home order.

We compared residuals, defined as the observed counts in 2020 minus the predicted counts for 2020, based on the prediction models we created for each counter from 2019 data (or 2018 data for New York City), across time periods. We ran t-tests comparing the mean residuals in the pre-Stay Home order period to those in the Stay Home order period. This was done to assess for a change in daily bicycle counts and a change in daily pedestrian counts between the pre-Stay Home order and Stay Home order periods. Note that the normality assumption of t-tests generally held across counter locations according to Q-Q plots. We also tested the significance of differences using the Wilcoxon Rank Sum test, which nonparametrically yielded the same conclusions. In Seattle, we had data available to compare mean residuals across the pre-Stay Home order, Stay Home order, and post-Stay Home order periods, applying Tukey's range test to assess for changes in the mean residuals between periods. Theoretically, the residuals mainly reflect the effects of COVID-19 and any other effects of uncontrolled extraneous variables. In other words, if the controlled extraneous variables were to explain all the variance in daily counts, then the residuals would be zeros. In the case that the mean residuals in the Stay Home order period were significantly larger (smaller) than those in the pre-Stay Home order period, the counts in the Stay Home order period increased (decreased) significantly from what the model would have predicted had there been no Stay Home order. In all cases, we used the threshold of p<0.05 to determine statistical significance.

## Results

Across all three cities and nearly all counter locations, we found a significant change in bicycle and pedestrian counts from the pre-Stay Home order period to the Stay Home order period (Table 2). The direction of change varied across the three cities as well as within cities, likely due to differing local contexts, different street and trail use types, local restrictions, and outbreak progression. Table 2 shows the differences in mean residuals across the pre-Stay Home order and Stay Home order periods at all counters across the three cities and the post-Stay Home order residuals at the Seattle counters. All three cities saw a significant change in mean residuals from the pre-Stay Home order period to the Stay Home order period at most counters included in the analysis (Table 2).

**Table 2:** Pre-Stay Home order, Stay Home order, and post-Stay Home order mean observed minus predicted daily bicycle or pedestrian counts by location.

Location	Pre- Stay Home order^1^ mean residuals	Stay Home order^2^ mean residuals	Post- Stay Home order^3^ mean residuals	Difference in means^4^, during to pre	Difference in means, post to during	Difference in means, post to pre
**Bicycle counts**						
**Houston**						
Brays Bayou Greenway	21.6	119.7	-	98.1 (62.3, 133.9)***	-	-
White Oak Bayou	55.3	248.5	-	193.2 (114.8, 271.5)***	-	-
Woodway Dr	18.3	226.8	-	208.5 (172.0, 244.8)***	-	-
**New York City**						
Brooklyn Bridge	490.2	-1462.4	-	-1,952.6 (-2,465.8, -1,439.4)***	-	-
Kent Avenue	2,003.3	998.5	-	-1,004.8 (-1,777.6, -231.9)**	-	-
Prospect Park West	3,837.1	1,029.7	-	-2,807.3 (-3,562.4, -2,052.3)***	-	-
Pulaski Bridge	1,527.8	1,106.2	-	-421.6 (-1,089.4, 246.2)	-	-
Williamsburg Bridge	7,540.5	1,116.5	-	-6,424.0 (-8,118.2, -4,729.8)***	-	-
**Seattle**						
Burke Gilman at 70th NE	1.1	390.5	143.4	389.4 (220.7, 558.2)***	-247.1 (-411.6, -82.6)**	142.3 (-15.2, 299.8)
Elliott Bay Trail	48.7	17.5	-265.0	-31.2 (-198.9, 136.5)	-282.5 (-445.9, -119.0)***	-313.7 (-470.2, -157.2)***
Fremont Bridge	53.5	-3,457.1	-6,113.1	-3510.6 (-4336.2, -2684.9)***	-2656.1 (-3460.8, -1851.3)***	-6166.6 (-6937.2, -5396.0)***
MTS Trail at I-90	-52.1	131.7	-95.6	183.8 (81.1, 286.5)***	-227.3 (-365.0, -89.5)***	-43.5 (-178.2, 91.2)
**Pedestrian counts**						
**Houston**						
Brays Bayou Greenway	-19.3	-21.0	-	-1.7 (-9.9, 6.5)	-	-
White Oak Bayou	741.6	1,246.9	-	505.3 (-49.9, 1060.4)	-	-
Woodway Dr	-0.70	73.1	-	73.8 (46.1, 101.5)***	-	-
**Seattle**						
Burke Gilman at 70th NE	40.1	258.5	108.7	218.4 (176.3, 260.6)***	-149.8 (-190.9, -108.7)***	68.6 (29.3, 108.0)***
Elliott Bay Trail	-528.7	-24.5	-1,386.7	504.2 (166.3, 842.1)**	-1362.2 (-1691.5, -1032.8)***	-858.0 (-1173.3, -542.6)***

^1^The pre-Stay Home order period corresponds to January 1, 2020 through the day prior to that state's Stay Home order.

^2^The Stay Home order period corresponds to the date of the Stay Home order through the end of the Stay Home order (May 31, 2020 in Seattle and June 12, 2020 in New York City) or the end of the available data (May 31, 2020 in Houston) June 30, 2020 in New York City and Seattle, and May 31, 2020 in Houston.

^3^The post-Stay Home order period corresponds to the date the Stay Home order was lifted through the end of the available data (these data are only available for Seattle, June 1, 2020 to August 31, 2020).

^4^The reported difference in means corresponds to the post-period mean residual minus the pre-period mean residual.

***p < 0.001

**p < 0.01

*p < 0.05

**Fig 2:**
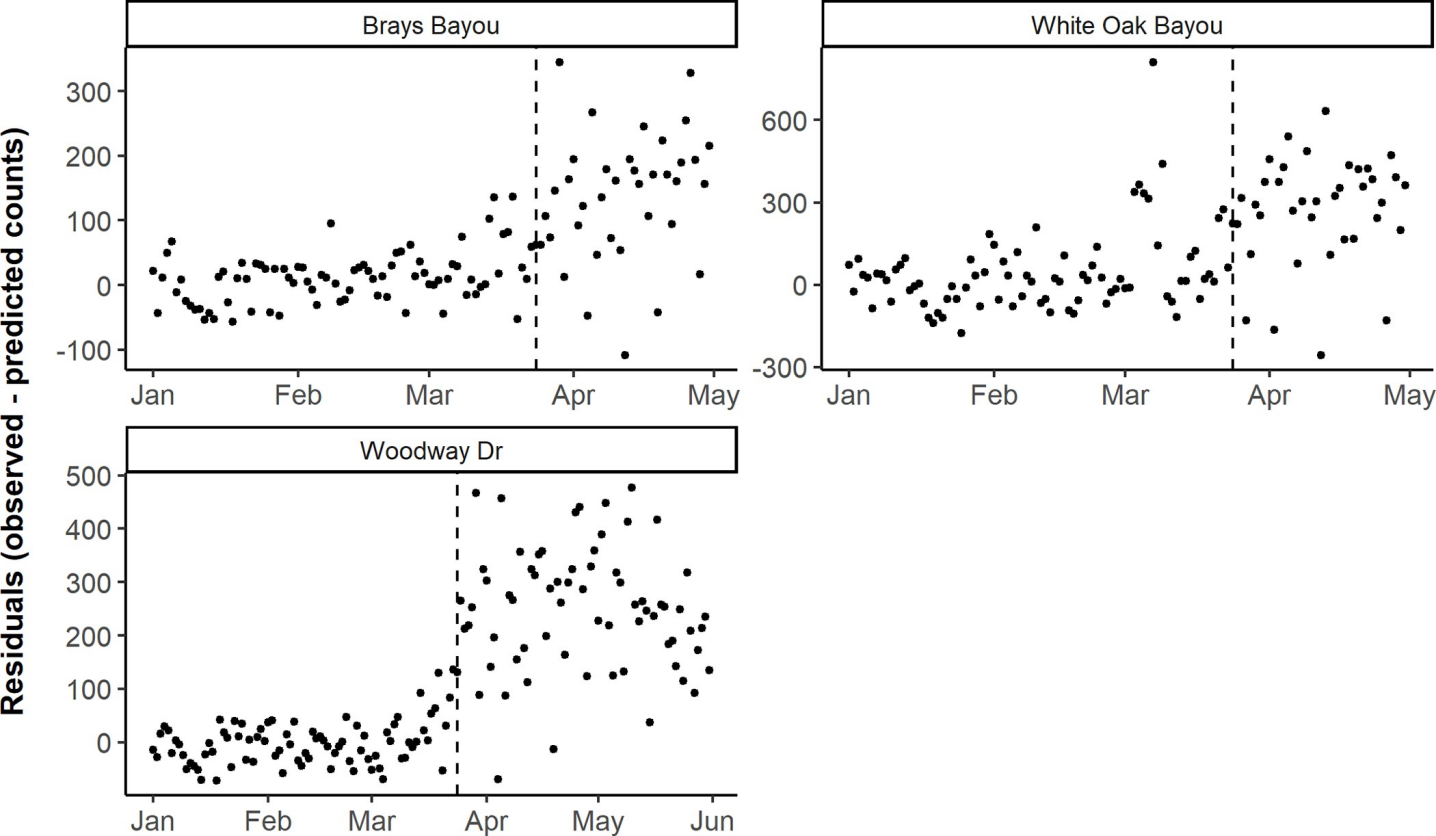
Daily observed minus predicted bicycle counts by counter location, before and during the Stay Home order in Houston. The vertical dashed line corresponds to the Stay Home order on March 24, 2020 in Harris County, Texas. Note the y-axis scales vary between plots to facilitate the interpretation of results.

**Fig 3:**
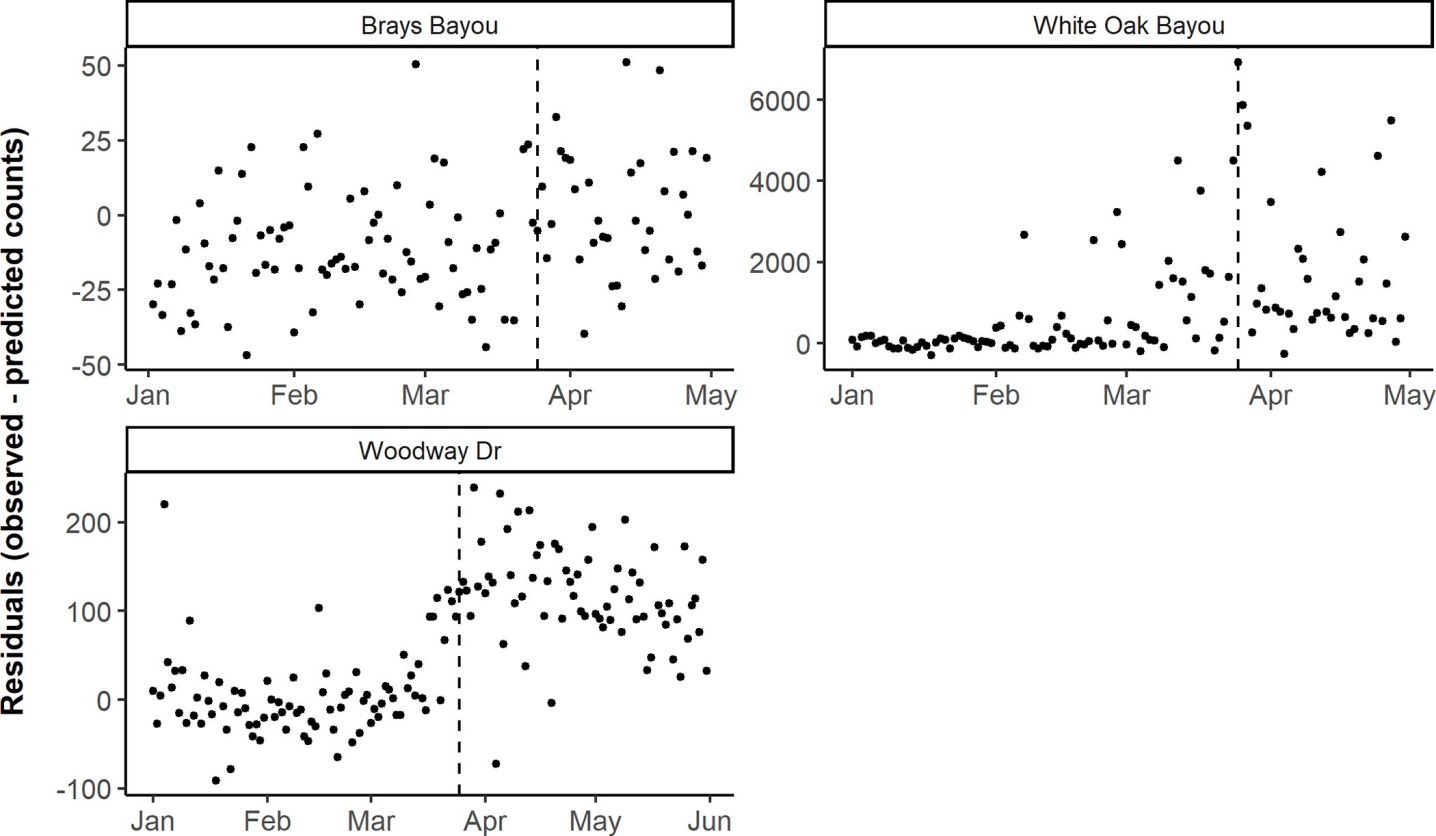
Daily observed minus predicted pedestrian counts by counter location, before and during the Stay Home order in Houston. The vertical dashed line corresponds to the Stay Home order on March 24, 2020 in Harris County, Texas. Note the y-axis scales vary between plots to facilitate the interpretation of results.

In Houston, bicycle counts saw a significant increase in mean residuals between the two periods across all three locations (p < 0.001), indicating that daily bicycle counts were significantly higher during the Stay Home order than predicted from the 2019 prediction model and 2020 covariate data (Fig 2 and S2 Fig). For pedestrian counts, one location in Houston saw virtually no change in mean residuals across the two periods, while two saw an increase, although only one was significant (p < 0.001) (Fig 3 and S3 Fig).

**Fig 4:**
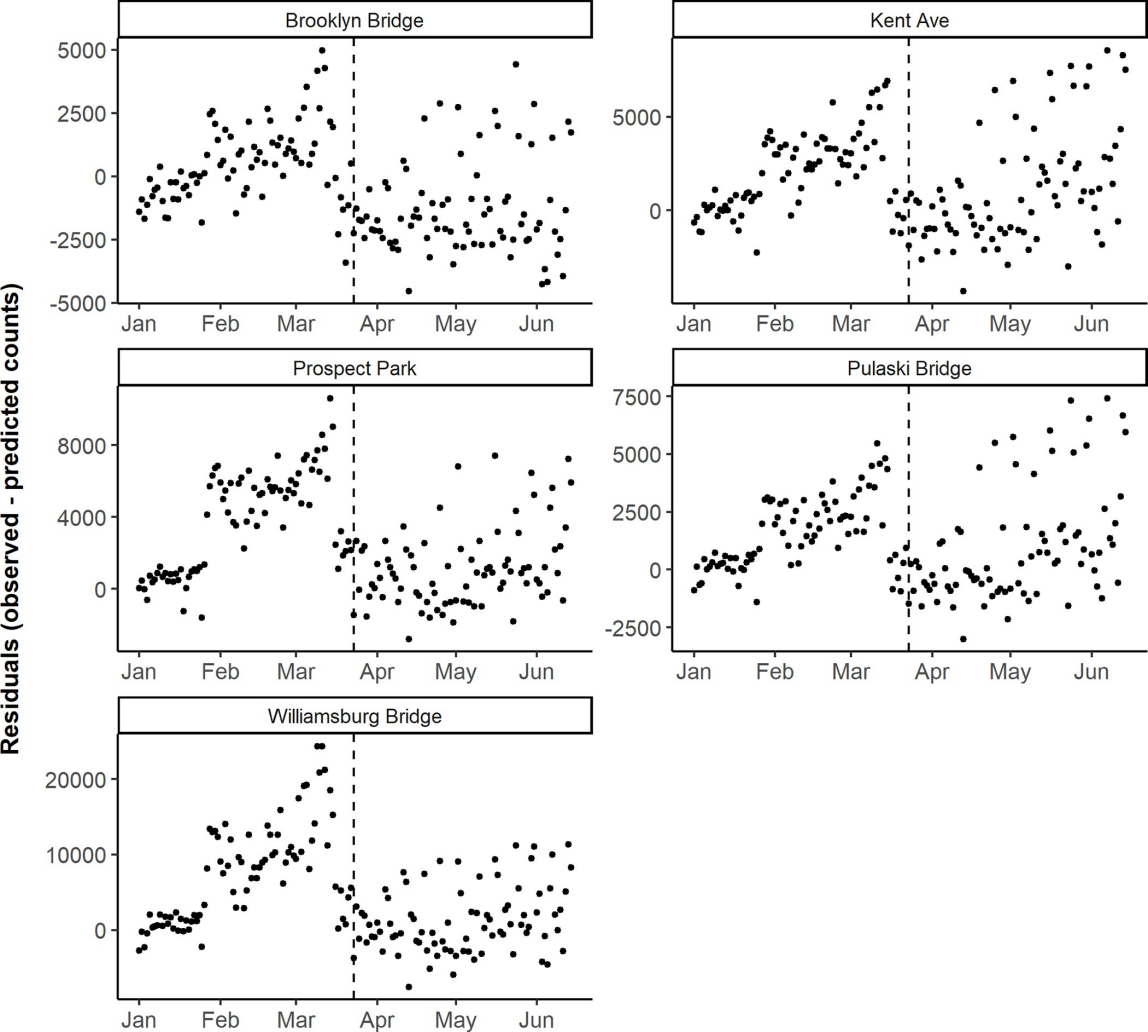
Daily observed minus predicted bicycle counts by counter location, before and during the Stay Home order in New York City. The vertical dashed line corresponds to the Stay Home order on March 22, 2020 in New York State. Note the y-axis scales vary between plots to facilitate the interpretation of results.

The trends in New York City bicycle counts appear relatively unique among the three cities in this analysis. All five counters saw a decrease in mean residuals from the pre-Stay Home order period to the Stay Home order period (Table 2), with four that were significant (p < 0.01). New York City experienced an increase in outdoor physical activity before the Stay Home order compared to predictions, as noted in a New York Times article from March 2020 [14]. This was followed by a marked decrease in daily bicycle counts at the beginning of the Stay Home order, corresponding to the surge of COVID-19 cases in New York City. Following this was a slow return to higher activity levels (Fig 4 and S1 Fig).

**Fig 5:**
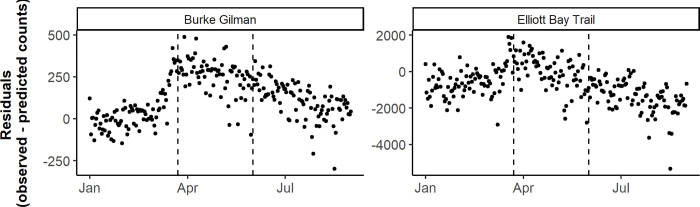
Daily observed minus predicted pedestrian counts by counter location, before, during, and after the Stay Home order in Seattle. The first vertical dashed line corresponds to the Stay Home order on March 23, 2020, and the second vertical dashed line corresponds to the end of the Stay order on May 31, 2020 in Washington state. Note the y-axis scales vary between plots to facilitate the interpretation of results.

**Fig 6:**
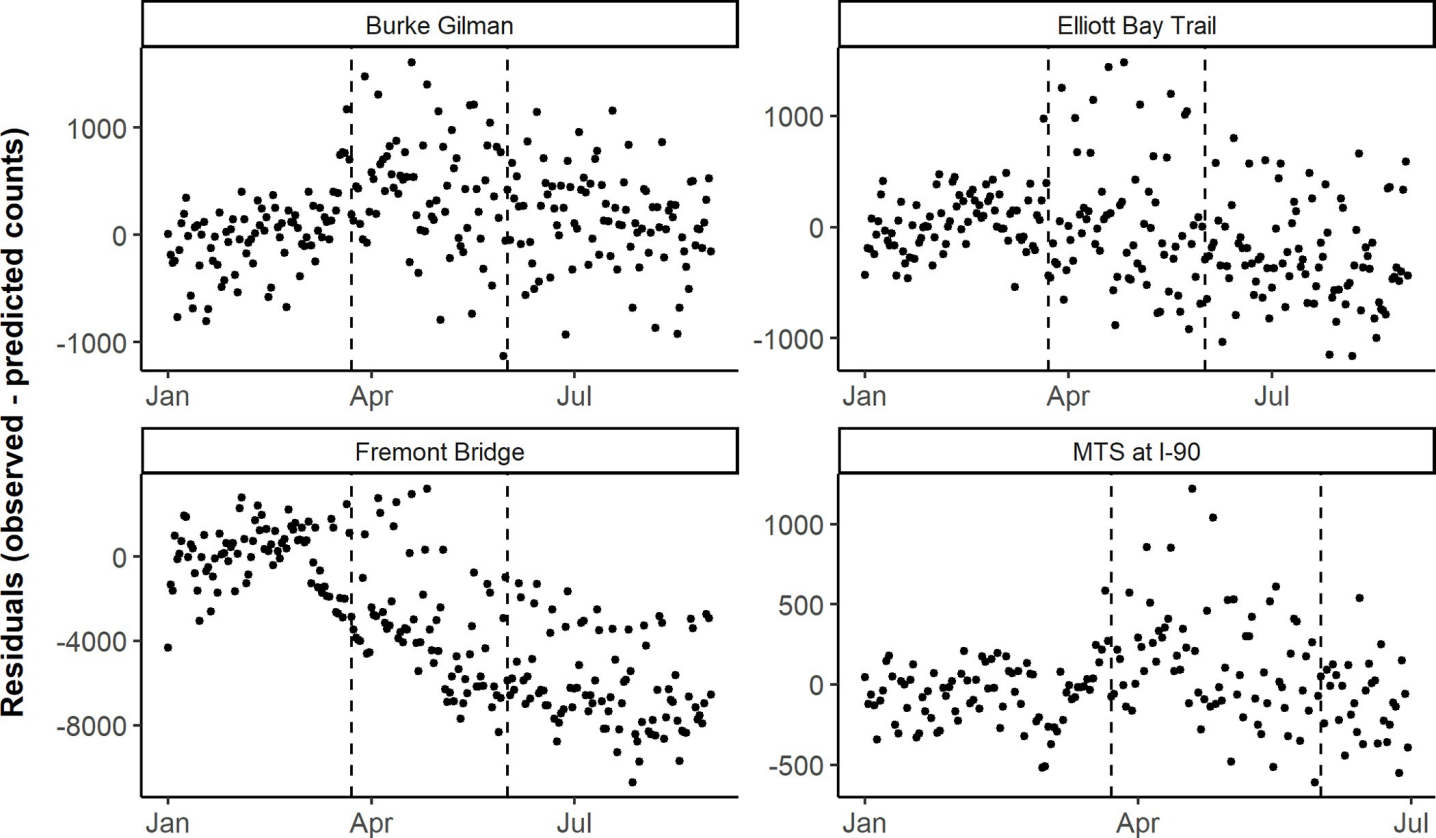
Daily observed minus predicted bicycle counts by counter location, before, during, and after the Stay Home order in Seattle. The first vertical dashed line corresponds to the Stay Home order on March 23, 2020, and the second vertical dashed line corresponds to the end of the Stay order on May 31, 2020 in Washington state. Note the y-axis scales vary between plots to facilitate the interpretation of results.

In Seattle, among bicycle counts, two counters saw a significant increase in mean residuals from the pre-Stay Home order to the Stay Home order period (p < 0.001), and two saw a decrease in mean residuals across the two periods, although only one was significant (p < 0.001) (Table 2). The results of Tukey's range test showed a significant decrease in mean residuals in the period following the Stay Home order compared to the Stay Home order period (p < 0.01). Three of four counters saw a decrease in mean residuals from the post-Stay Home order period compared to the pre-Stay Home order period, of which two were significant (p < 0.001). Among pedestrian counts, the results of Tukey's range test for the two counters with pedestrian data (Burke Gilman and Elliott Bay Trail) showed similar trends to the bicycle data (Table 2, Fig 5 and S4 Fig), with a significant increase in mean residuals during the Stay Home order compared to the pre-Stay Home order period (p < 0.001), a significant decrease in the post-Stay Home order period compared to the Stay Home order period (p < 0.001), and one counter with a significant increase (p < 0.001) and the other with a significant decrease (p < 0.001), comparing the post-Stay Home order to the pre-Stay Home order period. In Seattle, differing trends emerged based on the use type of the bicycle and pedestrian path. The areas by the Elliott Bay and Fremont counters are common areas for urban walks, and both saw less bicycle and pedestrian activity than predicted during the Stay Home order period. The other two areas, the Burke Gilman Trail and Mountain to Sound trail are more commonly used for recreation. Both counters saw more bicycle and pedestrian activity than predicted during the Stay Home order period (Table 2, Figs 5 and 6, and S5 Fig).

## Discussion

In this exploratory study, we aim to examine how Stay Home orders in three U.S. cities impact outdoor physical activity. To our knowledge, this analysis is the first of its kind to leverage daily bicycle and pedestrian count data to assess the impact of restrictive public health measures on physical activity during a pandemic. Our results indicate a significant change in outdoor physical activity behavior during the Stay Home orders compared to the period prior that varied by location, likely based on local context and trail use type. In Houston, nearly all locations saw significantly more daily bicycle and pedestrian counts during the Stay Home order period than predicted compared to the period before. In New York City, all locations reported significantly fewer daily bicycle counts during the Stay Home order period than predicted compared to the period before. And in Seattle, the results varied by trail use type, with two counters showing significantly more daily bicycle and pedestrian counts than predicted during the Stay Home order period compared to the period before, and two counters reporting fewer counts than predicted compared to the period before. The trends in Seattle during the period after the Stay Home order was lifted indicate a return to predicted levels of outdoor physical activity, although this varied by trail use type.

The differing results shown across the three cities likely reflect the reality of the situation on the ground in each region. There is a clear increase in daily bicycle counts in Houston compared to predicted daily counts during the Stay Home order period. This may be due, in part, to the relatively smaller outbreak in the region at the time, corresponding to less restrictive policies during the earlier months of the pandemic in Houston [16], compared to other regions of the U.S. In New York City, we see an increase in physical activity compared to predictions much earlier than in Houston and Seattle, possibly due to people seeking alternate modes of transportation more than a month before the Stay Home order to avoid the crowded subway system [14]. Finally, in Seattle, we find variability at counter locations within the city, possibly attributed to different use types of the trails (commuter versus recreational) and possible impacts from park closures at the city and state levels. However, more work is needed to understand the specific impacts at the local level.

The literature published thus far on physical activity during the COVID-19 pandemic generally finds a decrease in self-reported physical activity following Stay Home orders [6, 9], with some studies reporting variability by country and individual pre-pandemic activity level [6, 7]. However, our findings show differing changes in physical activity in the period immediately following Stay Home orders by location, suggesting that physical activity changes may differ in part due to local context. Additionally, we see evidence of changing physical activity behavior over time, likely in response to local policies, outbreak severity, messaging, and individual perception of risk, among other factors.

Physical activity [1] and outdoor time [2] are critical to wellbeing, particularly when most indoor community and recreational spaces are closed. Despite inconsistencies in trends across cities, our results show that use of trails was ubiquitous following Stay Home orders, demonstrating demand for access to protected public spaces in the context of pandemics. Urban areas may respond with policies and programs that encourage safer outdoor physical activity and expand access to such spaces to groups who might not live in proximity to an existing trail. For example, in Seattle, the city opened several miles of residential streets for use by bicycles and pedestrians to encourage safe outdoor physical activity during the pandemic, as well as to mitigate overcrowding seen in several city parks earlier in the outbreak [17]. Similarly, New York City’s Open Streets Program closed 100 miles of streets to improve physical distancing. While the program initially included streets that were closed for “play,” this program appears to have been seasonal (reported to have ended when this website was accessed in November 2020) [18].

At the same time, we observed no trails with extreme spikes in usage following Stay Home orders. However, several cities including Seattle and Houston closed parking lots at public parks to dissuade overcrowding [19, 20]. Given the importance of physical activity for physical and emotional health, cities may consider promoting non-park public spaces, including trails, as alternatives to parks to encourage physical activity while adhering to physical distancing guidelines.

Our analysis is limited by the lack of individual-level data. For example, our data may be showing a shift from indoor physical activity to outdoor activity among the already physically active. More reliable individual-level data are needed to better understand how disasters and restrictive public health measures in the COVID-19 pandemic impact community physical activity, given its cascading impacts on health and well-being. Furthermore, these data may not be representative of each city's physical activity patterns on the population level.

These results can begin to inform local-level policy making around the use of outdoor public infrastructure as the COVID-19 pandemic continues across the U.S. Given the generally higher than normal, yet not extreme use of outdoor public infrastructure demonstrated here, policymakers should consider strategies, including those discussed above, to meet the demand for physical activity without overcrowding outdoor public spaces during a pandemic.

## Supporting information

S1 Fig Daily bicycle counts by location in New York City, before and during the Stay Home order. The vertical dashed line corresponds to the Stay Home order on March 22, 2020 in New York State. Note the y-axis scales vary between plots to facilitate the interpretation of results.
(DOCX)

S2 Fig Daily bicycle counts by counter location in Houston, before and during the Stay Home order. The vertical dashed line corresponds to the Stay Home order on March 24, 2020 in Harris County, Texas.
(DOCX)

S3 Fig Daily pedestrian counts by counter location in Houston, before and during the Stay Home order. The vertical dashed line corresponds to the Stay Home order on March 24, 2020 in Harris County, Texas. Note the y-axis scales vary between plots to facilitate the interpretation of results.
(DOCX)

S4 Fig Daily pedestrian counts by location in Seattle, before, during, and after the Stay Home order. The first vertical dashed line corresponds to the Stay Home order on March 23, 2020, and the second vertical dashed line corresponds to the end of the Stay order on May 31, 2020 in Washington state. Note the y-axis scales vary between plots to facilitate the interpretation of results.
(DOCX)

S5 Fig Daily bicycle counts by location in Seattle, before, during, and after the Stay Home order. The first vertical dashed line corresponds to the Stay Home order on March 23, 2020, and the second vertical dashed line corresponds to the end of the Stay order on May 31, 2020 in Washington state. Note the y-axis scales vary between plots to facilitate the interpretation of results.
(DOCX)
