# Supplementary material for: How did outdoor biking and walking change during COVID-19?: A case study of three U.S. cities
Source: PLoS One. 2021 Jan 20;16(1):e0245514. doi: 10.1371/journal.pone.0245514 (PMC7816985; doi:10.1371/journal.pone.0245514)
Supplement: S1 Fig — The vertical dashed line corresponds to the Stay Home order on March 22, 2020 in New York State. Note the y-axis scales vary between plots to facilitate the interpretation of results. (DOCX) [file pone.0245514.s001.docx]

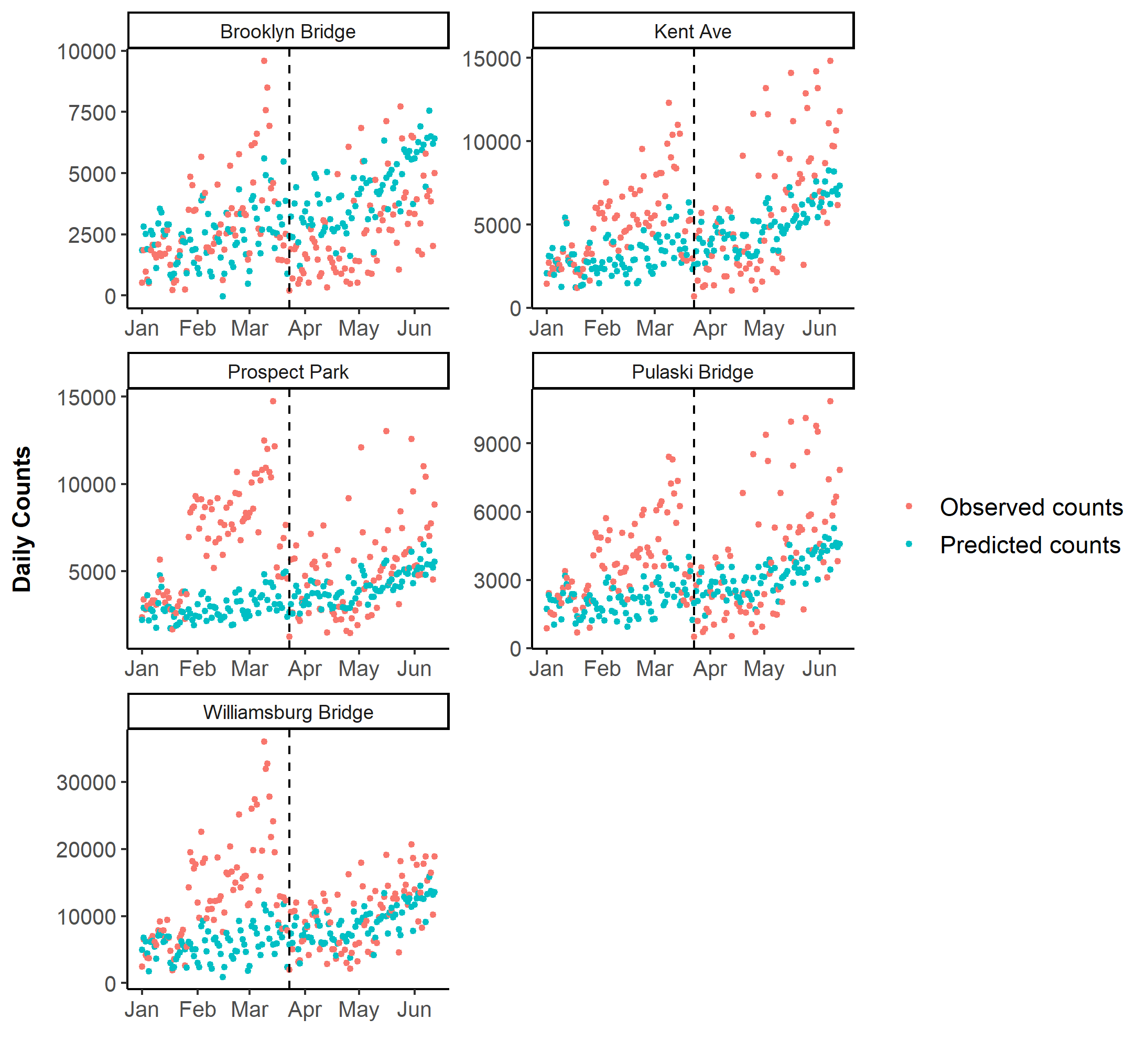


**S1 Fig.** Daily bicycle counts by location in New York City, before and during the Stay Home order. The vertical dashed line corresponds to the Stay Home order on March 22, 2020 in New York State. Note the y-axis scales vary between plots to facilitate the interpretation of results.
