# Supplementary material for: How did outdoor biking and walking change during COVID-19?: A case study of three U.S. cities
Source: PLoS One. 2021 Jan 20;16(1):e0245514. doi: 10.1371/journal.pone.0245514 (PMC7816985; doi:10.1371/journal.pone.0245514)
Supplement: S3 Fig — The vertical dashed line corresponds to the Stay Home order on March 24, 2020 in Harris County, Texas. Note the y-axis scales vary between plots to facilitate the interpretation of results. (DOCX) [file pone.0245514.s003.docx]

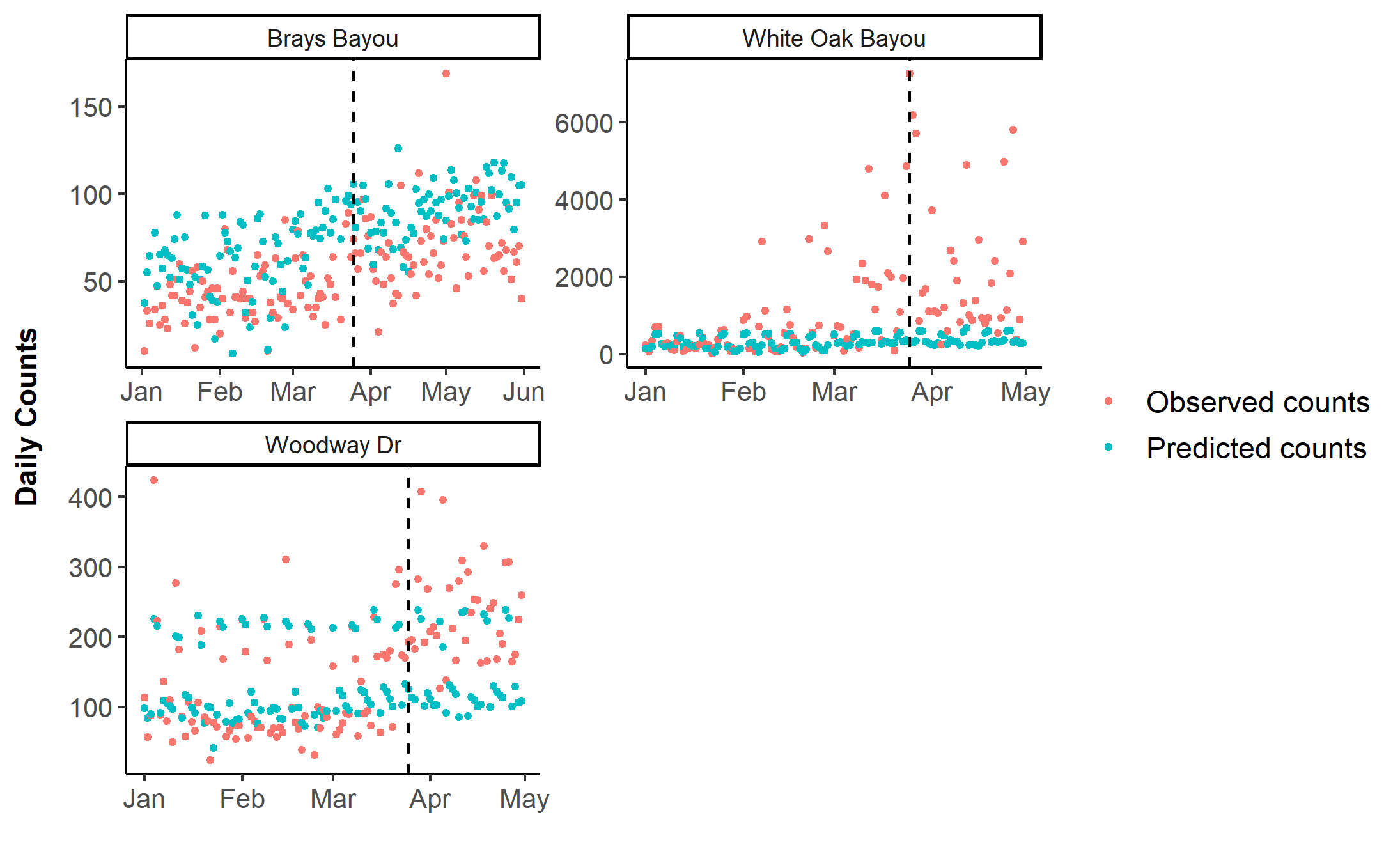


**S3 Fig.** Daily pedestrian counts by counter location in Houston, before and during the Stay Home order. The vertical dashed line corresponds to the Stay Home order on March 24, 2020 in Harris County, Texas. Note the y-axis scales vary between plots to facilitate the interpretation of results.
