# Supplementary material for: How did outdoor biking and walking change during COVID-19?: A case study of three U.S. cities
Source: PLoS One. 2021 Jan 20;16(1):e0245514. doi: 10.1371/journal.pone.0245514 (PMC7816985; doi:10.1371/journal.pone.0245514)
Supplement: S5 Fig — The first vertical dashed line corresponds to the Stay Home order on March 23, 2020, and the second vertical dashed line corresponds to the end of the Stay order on May 31, 2020 in Washington state. Note the y-axis scales vary between plots to facilitate the interpretation of results. (DOCX) [file pone.0245514.s005.docx]

**
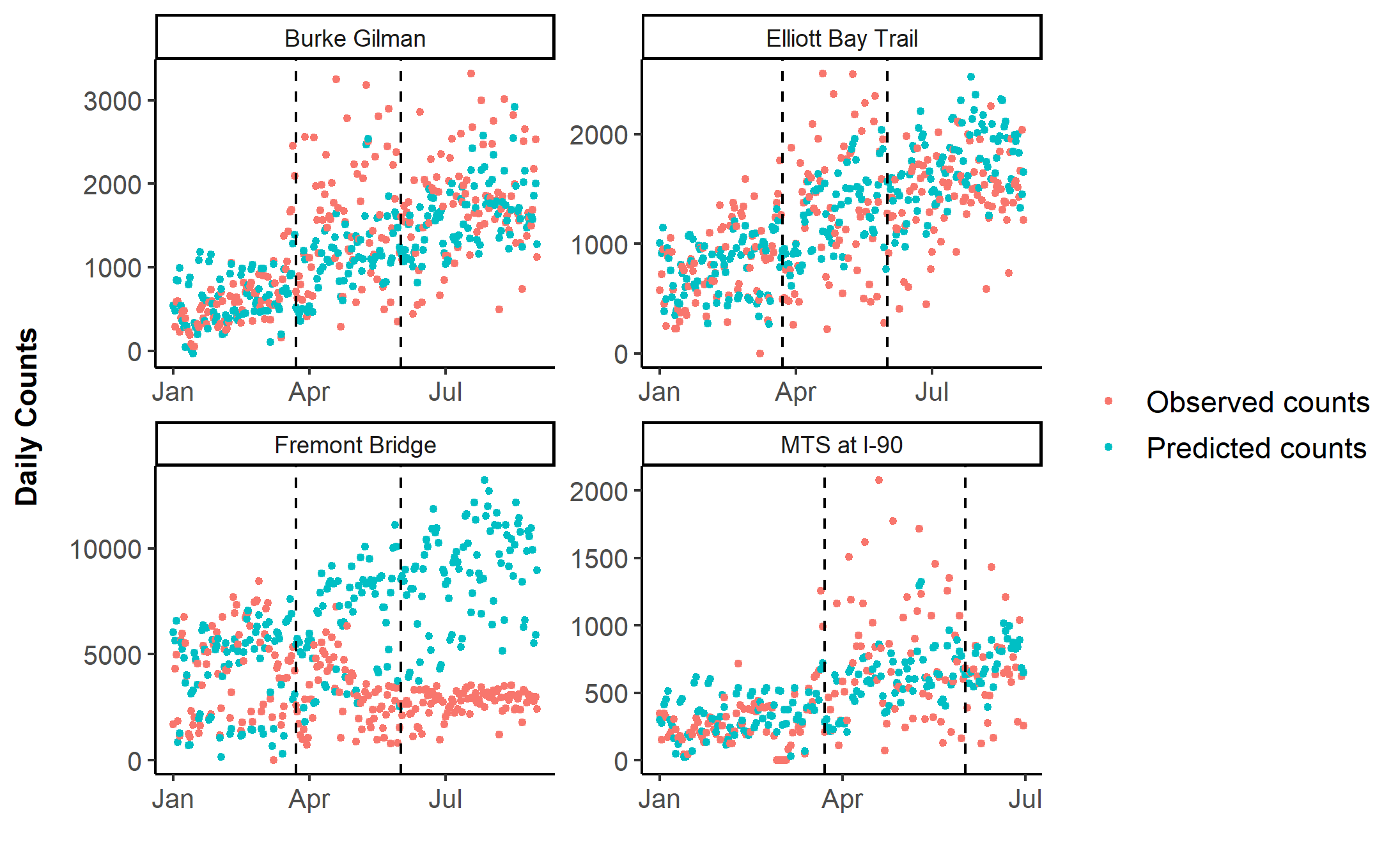
**

**S5 Fig.** Daily bicycle counts by location in Seattle, before, during, and after the Stay Home order. The first vertical dashed line corresponds to the Stay Home order on March 23, 2020, and the second vertical dashed line corresponds to the end of the Stay order on May 31, 2020 in Washington state. Note the y-axis scales vary between plots to facilitate the interpretation of results.
